# Prevalence of bucco-dental pathologies in patients with psychiatric disorders

**DOI:** 10.4317/jced.51147

**Published:** 2014-02-01

**Authors:** Mariana C. Morales-Chávez, Yusthin M. Rueda-Delgado, David A. Peña-Orozco

**Affiliations:** 1 Pediatric Dentist, Magister in Special Care Dentistry, Director of the Dental Research Center of Santa María University, Caracas, Venezuela; 2 Dentist, Santa María University, Caracas, Venezuela

## Abstract

Introduction: Oral diseases in psychiatric patients are usually a result of bad oral hygiene and psychopharmaceutical side-effects. Objective: The aim of this study was to detect the most prevalent oral lesions in patients hospitalized in a psychiatric institution in Caracas, Venezuela with the confirmed diagnosis of psychiatric illness. Material and Methods: A transversal study consisted of 65 hospitalized patients with psychiatric disorders out of whom 50 were males and 15 females. Patients were aged from 19 to 80 years, mean age 50.2 years. Data on oral lesions were obtained within history and clinical examination of the oral cavity. Other medical data were collected from medical documentation. Statistical analysis was performed by SPSS version 17.0. Results: 56.92% of patients had caries in at least one tooth, 29.23% presented gingivitis and 56.92% periodontal disease. In relation to Temporomandibular joint, 36.92% presented articular sounds and 10.76% muscular pain. Between the most prevalent parafunctional habits were found cigarette habit, bruxism, onychophagia and cheek bite. Conclusion: Results imply that psychiatric patients are more frequently involved with oral lesions than healthy persons. It is necessary to organize specific preventive and educational oral health programmes with these patients, in a multidisciplinary group.

** Key words:**Phychiatric patients, schizophrenia, medication, periodontal diseases.

## Introduction

Psychiatric disorders have become an increasingly frequent health problem mostly affecting the elderly, representing 10% of the global load of diseases, a figure that is expected to reach 15% by 2020. Health professionals should be increasingly interested in the managing of such pathologies (1).

Older adults have a higher propensity to develop painful pathologies and dementia. With the increase of life expectancy, there has been an exponential increase in the number of individuals affected by dementia in the last few years. Several studies have indicated an incidence of dementia of 1% between 60 and 65 years of age, values that double every five years, until reaching 25% to 50% at 85 years of age (2).

Mouth health is an integral part of the general health, and it affects all aspects of life: personal, social, and psychological. This is particularly important in patients with special needs, such as psychiatric patients (1). These patients have a tendency to be more prone to develop bucco-dental diseases, due to their lack of motivation, the difficulty to perform a proper mouth health technique, the hurdles that have to be overcome to treat them dentally, and the negative effects caused by psychotropic medications, affecting the normal physiology of salivary glands and epithelia of the oral mucous, causing xerostomy or sialorrhea (3-5).

Different authors have reported that patients with mental illnesses get inadequate dental care due to ignorance, fear, stigmas, or negative attitudes by the professionals (1).

Dental care in patients with mental disorders should be performed considering their systemic conditions, both of which, as already proved in the last two decades, are closely related. The care protocol for these patients will vary depending on factors such as the communication level that could be established with the patient, medical history, age, priorities in the dental treatment, prognosis, and future maintenance (1,6).

Patients suffering from chronic mental illness represent a risk group for mouth health. It has been demonstrated that psychiatric patients have a more frequent occurrence and severity of cavities, periodontal diseases, and lesions of the oral mucous (7).

Medications administered for the control of signs and symptoms of psychiatric pathologies have well known side effects, all of which the specialist should be fully aware of, so as to avoid negative effects, since in most cases it is impossible to change the medications or dosage. Sedatives and anxiolytics can cause cardiorespiratory depression, and in some patients they cause paradoxical reactions. The only interaction with benzodiazepines related to dentists are the macrolides (erythromycin), as their mix produces an increase in the levels of serum, and of the half-life (8).

## Material and Methods

An observational transversal study was carried out, in which 65 institutionalized patients were evaluated at the Psychiatric Center of the Venezuelan Social Security Institute, in Caracas. In the inclusion criteria, all patients with an informed consent signed by them or by their representative, accepting the dental evaluation, were considered, as well as the patients that were not aggressive when allowing the clinical test. Ethical approval for the study was obtained from the Ethical Committee of the Dental School of Santa Maria University. The ages were between 19 and 80 years, with a 50.2 average, and a 51 median. The distribution in terms of gender was 15 (23.07%) females, and 50 (76.9%) males. Once the sample was selected, the case histories of each patient were reviewed and the caretakers were questioned on the presence of habits, also data, such as psychiatric diagnosis and the medication each of them was getting, were collected. In the medical diagnosis, the following pathologies were observed: Schizophrenia, behavioral mental disorders, bipolarity, organic psychosis, and dementia. Finally, among the families of medications consumed by them, anxiolytics, antipsychotics, neuroleptics, anticonvulsants, and antiparkinsonians were found.

To collect saliva was used the suction method of non-stimulated saliva, 2 hours after breakfast to avoid food remainders. Normal non-stimulated salivary flow rate ranges from 0.3 to 0.5 mL/min, and flow rates between 0.10 and 0.01 mL/min were considered hyposalivation.

A single previously calibrated operator, evaluating the presence of cavities, periodontal disease, oral pathologies or temporomandibular joint disorders, examined all patients. The gingival status was assessed using the Löe-Silness Gingival Index (GI) and Periodontal status was determinated by Ramfjord Periodontal Disease Index. Statistical analysis was performed by SPSS version 17.0.

## Results

In the present study, 65 patients with psychiatric disorders, such as bipolarity (12.30%), psychosis (13.86%), dementia (10.76%), mental disorder (3.07%), and schizophrenia (60%), were evaluated ( Table 1).

**Table 1:**
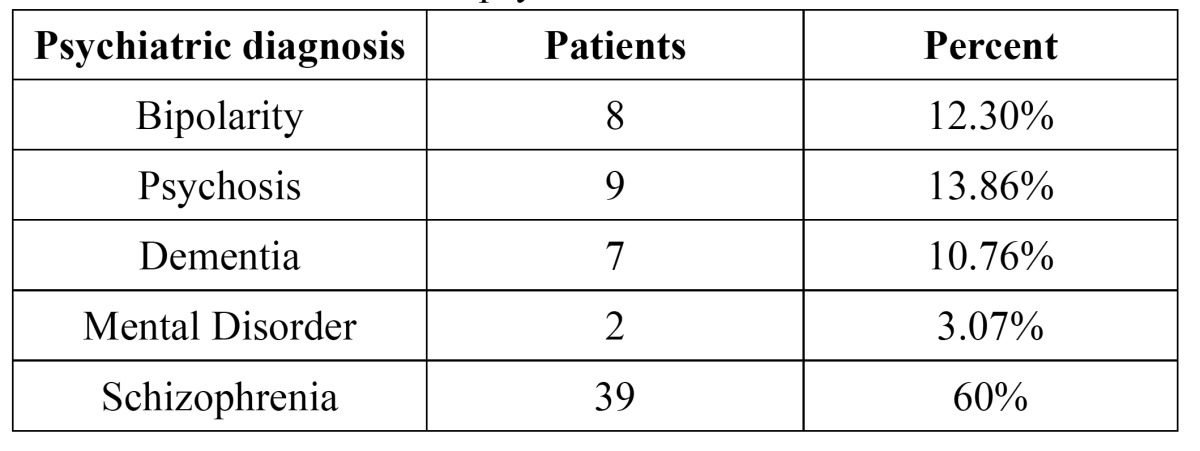
Distribution of the psychiatric disorders.

It was determined that 56.92% of the patients had cavities, and the remaining 43.08% did not have any lesions of this type, and that could be clinically observed. In terms of the periodontal disease, it was observed that 13.84% (9 patients) of the sample did not present any clinical signs of periodontal disease, unlike the 29.23% that was diagnosed with gingivitis, and 56.92% with periodontitis. In the oral mucous evaluation were diagnosed 1 patient (1.5%) with melanotic pigmentation and 2 patients (3.07%) with candidiasis that were diagnosed with a culture of C. albicans.

After saliva was measured was determinated that 9.2% of the patients presented xerostomy and 38.4% was diagnosed with hypersalivation.

In terms of the temporomandibular joint, 36.92% presented some joint noise. On the other hand, 26.15% of the sample presented a deviation in mandibular opening or closing. 26.15% of the patients had limitations in opening, and only 10.76% reported muscle pain on palpation ( Table 2).

The case history of each patient was evaluated, and the caretakers were questioned on the presence of habits and was determinated that 44.6% of the patients had habits. It was found that 6.15% had bruxism, 27.69% smoked, 1.53% smoked and also presented onychophagia and bruxism and 3.07% smoke-related bruxism, onychophagia and cheilophagia ( Table 3).

**Table 2:**
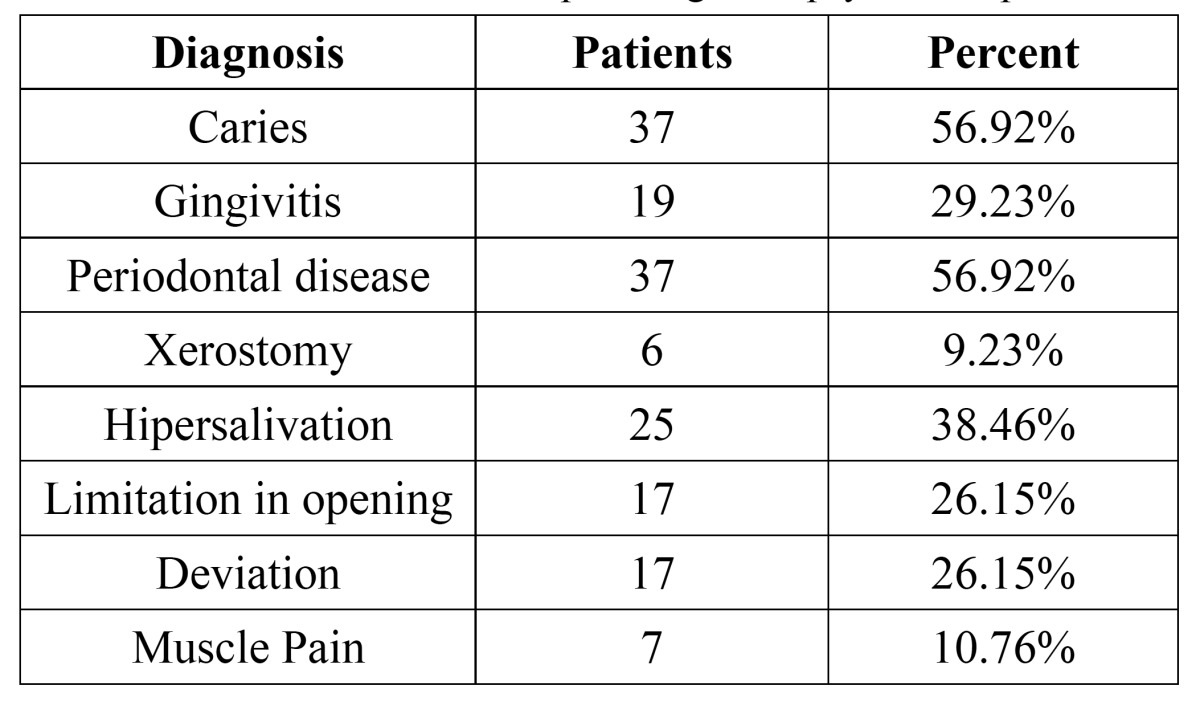
Prevalence of different pathologies in psychiatric patients.

**Table 3:**
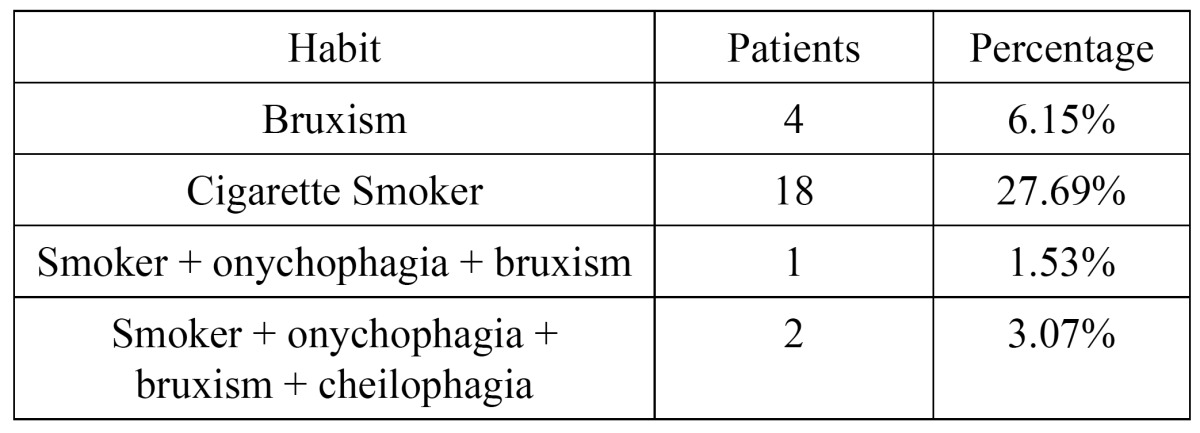
Different habits presented by the psychiatric patients.

In terms of medications, 100% of the sample was taking more than one medication. 24.2% (15 patients) was taking anxiolytics (diazepam); 78.46% (51 patients) anticonvulsants (trileptal, valproic acid, carbamazepine, clonazepam and valproate semisodium); 50.76% (33 patients) antipsychotics (olazapine, risperidone, periciatina and levomepromazine); 10.76% (7 patients) antihypertensives (enalapril, propanolo, atenolol and furosemide); 38.46% (25 patients) antiparkinsonians (bisperiden hydrochloride and levodopa); 56.92% (37 patients) neuroleptics (haloperidol and trifluoperazine) and 72.6% of the sample also consumed other types of medications like gastric mucosa protective agents and antiagregants ( Table 4).

**Table 4:**
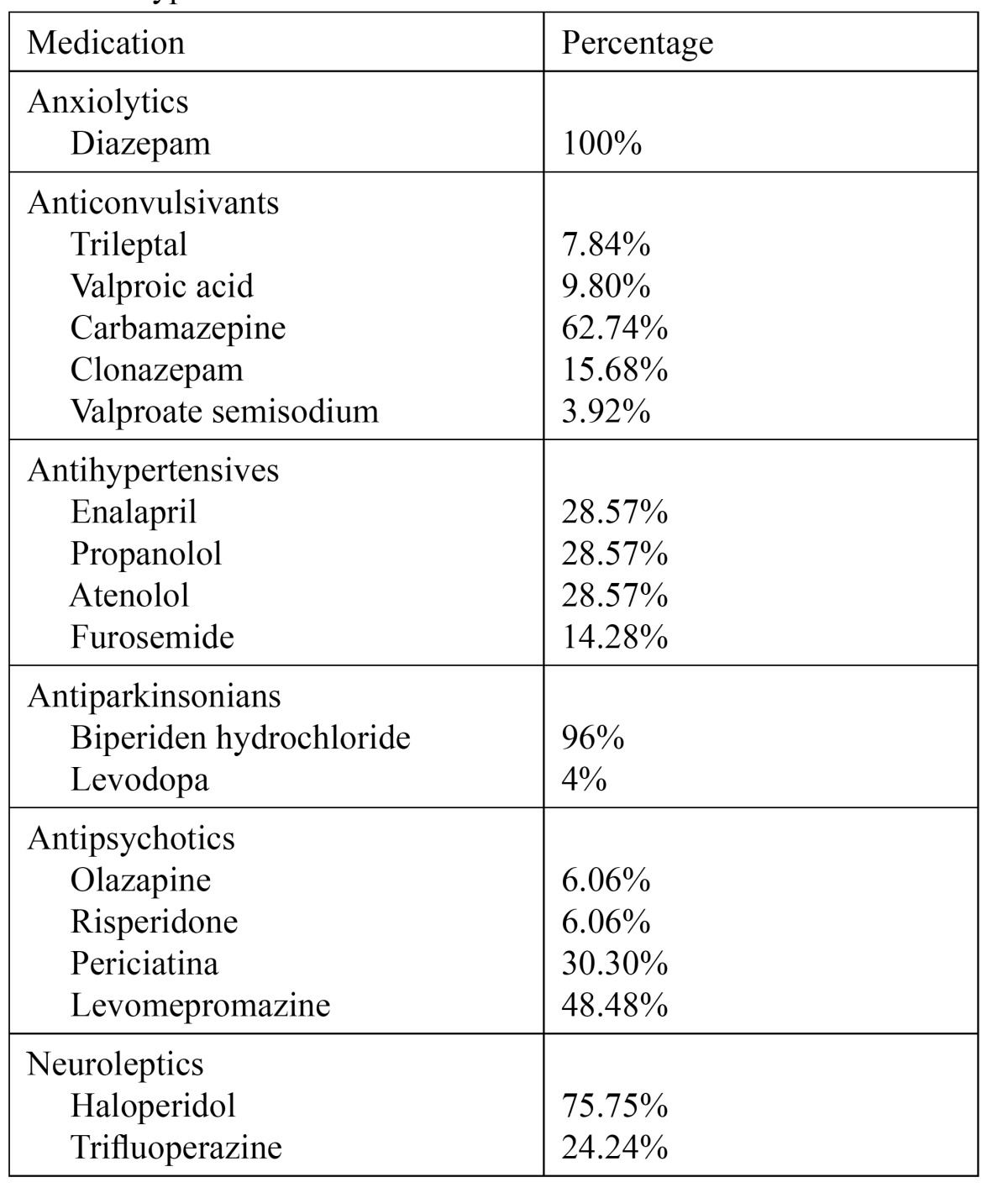
Types of medication.

## Discussion

Psychiatric patients, especially schyzophrenics, constitute a risk group for periodontal diseases. Psychiatric factors can affect the etiopathogeny of these periodontal disorders. Dental hygiene is usually poor, with higher occurrence of plaque, and stones. The periodontopathogenic plaque is more aggressive and adhesive, as a consequence of xerostomy and smoking (7). Clinical findings of this study proved that 29.23% of the patients presented gingivitis, 56.92% had periodontitis, and only 13.84% of the sample did not show any signs of periodontal disease. It is important to note that 78.46% of the patients were on anticonvulsants, which represents a risk factor for the development of periodontal diseases. Other studies, such as the one made by Gurbuz et al. (9) in Turkey, where 330 hospitalized patients with chronic psychiatric disorders were evaluated, concluded that only 8.8% of the patients presented healthy periodontal tissues. 6.3% presented gingival hemorrhage, 51.8% of the subjects had stones, and 33% deep periodontal sacs. In the same manner, Jovanovic et al. (10) evaluated 186 psychiatric patients, and 186 controls in Serbia, and determined that 3.3% had gingival hemorrhage, 15% dental stones, and 28.3% deep sacs. Only 8.3% did not show any sign of periodontal disease. Another study carried out by Eltas et al. (11), after performing a study with 53 psychiatric patients, with a medication which side effect was xerostomy or sialorrhea, concluded that there is a high risk for the development of periodontal disease in schizophrenic patients, and that such risk is even higher in patients that take a medication that causes a decrease in the flow of saliva. In terms of cavity indexes, 56.92% of the sample showed cavities, and 43.08% did not have any lesions of this kind. Similar indexes were obtained by Patel et al. (12), after the evaluation of 112 patients with mental disease, and determined that 53% of them had cavities in at least one of the teeth. Likewise, Kossioni et al. (13), after studying a sample of 11 hospitalized psychiatric patients, determined that 50.7% of them showed at least one cavity.

The importance of psychiatric disorders associated to temporomandibular disorders (TMD) has been mentioned in the literature, showing a relationship in the appearance, clinic, prognosis, and treatment of TMDs with psychosocial factors, such as stress, anxiety, and depression (7). In terms of the temporomandibular joint, 36.92% presented some joint noise. Velasco-Ortega et al. (7) studied a sample of 50 schizophrenic patients, and 50 controls, and determined that 24% of schizophrenic patients presented joint noises. In the present study, 26.15% of the sample showed a deviation in mandibular opening or closing, and 10.76% reported muscle pain on palpation. Most clinical studies that compare their sample to control groups have been able to determine significant statistical differences, where study groups show a higher occurrence of temporomandibular disorders than in healthy patients. Liao et al. (14) studied a sample of patients suffering from depression, and a control group, and concluded that the occurrence of temporomandibular disorders was 2.65 times higher in depressed patients.

Evaluation of the oral mucous in studied patients only produced 2 patients (3.07%) with candidiasis, and 1 (1.5%) with melanotic pigmentation. However, authors like Dangore-Khasbage et al. (1), after evaluating the prevalence of lesions of the oral mucous in psychiatric patients, determined that the most common were aphthous stomatitis, burning mouth syndrome, and lichen planus, followed by xerostomy, dysgeusia, frictional keratosis, bruxism, and cheilophagia.

Previous studies have suggested that the use of drugs for psychiatric treatment is a predisposing factor for xerostomy. Many psychotopics, such as antipsychotics, benzodiazepines, mood stabilizers, and antidepressants, cause xerostomy because they interfere with the function of the salivary glands. Nevertheless some antipsycotics like Risperidona increase the saliva’s production (1).

The results of this study concluded that only 9.2% of the patients presented xerostomy. However, 38.4% was diagnosed with hypersalivation.

Friedlander et al. (15) reported a detailed study of the effects of psychotropic medications on the buccal mucosa. It relates the antipsychotic agents with the occurrence of dysgeusia, xerostomy, and stomatitis; benzodiazepines with xerostomy and sialorrhea; mood stabilizers and lithium with xerostomy, and dysgeusia, and carbamazepine with xerostomy and glossitis.

Psychiatric disorders are a public health problem growing every day in larger proportions. Mouth health of these patients is usually more affected than the rest of the population, as mouth hygiene is more complicated, they do not get enough dental care, and take several medications that affect the flow of saliva, and the buccal mucosa. For this reason, the risk of suffering from buco-dental diseases is very high, and thus it is necessary to create preventive and educational buccal health programs for psychiatric patients, comprised by a cross-disciplinary team in which the physicians treating the base disease play a fundamental role.
